# Polystyrene nanoplastics differentially influence the outcome of infection by two microparasites of the host *Daphnia magna*

**DOI:** 10.1098/rstb.2022.0013

**Published:** 2023-03-27

**Authors:** Florent Manzi, Paula Schlösser, Agata Owczarz, Justyna Wolinska

**Affiliations:** ^1^ Department of Evolutionary and Integrative Ecology, Leibniz Institute of Freshwater Ecology and Inland Fisheries, 12587 Berlin, Germany; ^2^ Department of Biology, Chemistry, Pharmacy, Institute of Biology, Freie Universität Berlin, 14195 Berlin, Germany

**Keywords:** co-infection, *Metschnikowia*, *Ordospora*, plastic pollution, zooplankton

## Abstract

The accumulation of micro- and nanoplastic particles in freshwater bodies has given rise to much concern regarding their potential adverse effects on aquatic biota. Beyond their known effects on single species, recent experimental evidence suggests that host–parasite interactions can also be affected by environmental concentrations of micro- and nanoplastics. However, investigating the effects of contaminants in simplified infection settings (i.e. one host, one parasite) may understate their ecological relevance, considering that co-infections are common in nature. We exposed the cladoceran *Daphnia magna* to a fungal parasite of the haemolymph (*Metschnikowia bicuspidata*) and a gut microsporidium (*Ordospora colligata*), either in single or co-infection. In addition, *Daphnia* were raised individually in culture media containing 0, 5 or 50 mg l^−1^ of polystyrene nanoplastic beads (100 nm). Only few infections were successful at the higher nanoplastic concentration, due to increased mortality of the host. While no significant effect of the low concentration was detected on the microsporidium, the proportion of hosts infected by the fungal parasite increased dramatically, leading to more frequent co-infections under nanoplastic exposure. These results indicate that nanoplastics can affect the performance of distinct pathogens in diverging ways, with the potential to favour parasite coexistence in a common zooplanktonic host.

This article is part of the theme issue ‘Infectious disease ecology and evolution in a changing world’.

## 1. Introduction

Plastic contamination of natural ecosystems is on the rise. In addition to plastic products naturally breaking down into smaller particles [1,2], there is increasing demand for industrially produced microplastics (i.e. ‘MPs’, with a size less than 5 mm; [3]), needed for example in the field of cosmetics [4] or the booming sector of nanotechnologies [5]. Current sewage treatment methods cannot remove microplastics from wastewater [6] and novel remediation techniques still require further development to ensure the proper clearing of contaminated sites [7,8]. As such, increasing quantities of MPs accumulate in soil and aquatic compartments. The smallest defined fraction of MPs (commonly referred to as nanoplastics or ‘NPs’) comes in a variety of shapes and forms, with an upper size limit of 100 nm [9]. NPs can accumulate within living tissues [10], leading to their trophic transfer up the food chain [11,12]. Due to their small size, these can also penetrate living cell membranes [13,14], with the potential to disturb intracellular functions [15]. These inherent properties of NPs have raised great concerns in the scientific literature, notably due to their potential adverse effects on aquatic biota [16].

Owing to their status as a widely used model for ecotoxicological studies and their excellent suitability for advanced ecotoxicogenomics [17,18], zooplankters of the genus *Daphnia* have contributed to an impressive body of work on this matter. Over the course of the past decade, numerous publications have addressed the implications of microplastic particles [19,20] and, more recently, NPs [21–23] on the life-history and physiological responses of this freshwater model (reviewed in [24]). Besides frequent adverse effects on parameters of individual performance, such as longevity, body size and lifetime offspring production [14,25], typical responses of *Daphnia* to polystyrene NPs include enhanced production of reactive oxygen species, increased AMP kinase activity and direct changes in the levels of gene transcription, including stress defence-related loci [26–28]. Beyond the target-species approach used with *Daphnia*, as well as other organisms [29,30], experimental literature is still lacking information about the potential effects of NPs on interactions *between* species. While implications of plastic contamination have received some level of attention with regards to predator–prey interactions [12,31,32], studies investigating the impact of plastic pollution in the context of parasitic infections remain scarce. Recent advances have been made using other host species such as fruit flies [33], nematodes [34], amphibians [35] and phytoplankton [36], indicating that high concentrations of microplastic or NPs can strongly sway the outcome of parasitic infections. Surprisingly, despite the frequent use of *Daphnia* as a model of choice for host–parasite related studies (reviewed in [37,38]), no such attempt has been published on this genus.

Natural populations of *Daphnia* are exposed to a multitude of parasite species, some of which can reach very high prevalence [39–41] and individual hosts are often found bearing multiple parasites at once, sometimes in distinct compartments of their body [42]. Indeed, parasites of *Daphnia* cover a wide range of phylogenetic taxa, including Fungi, Microsporidia, Ichthyosporea, Bacteria [43–45] and a more recently identified viral pathogen [46]. These may differ widely in their primary strategies of infection, including modes of host-to-host transmission and the levels of virulence inflicted on the host [42,47,48]. Such discrepancies may also imply differential parasite responses to NPs: for instance, internalization of nanoparticles within microparasites could depend on their cell size, permeability (due to differing thickness and composition of the spores' cell wall), or their preferred site of establishment within the host (e.g. intra- or extracellular). Thus, the *Daphnia*-microparasite system represents a potentially excellent model to further uncover the ecological impact of microplastic and NP contamination.

Due to multi-species infections being common in natural populations of *Daphnia* [42] and more generally across eukaryotic biota [49,50], assessing the potential impacts of NPs using classical two-species systems (one host, one parasite) may not be sufficient. Instead, implementing more realistic conditions of frequent co-infections may be needed to accurately represent the ecosystem-wide intricacies of NP contamination. Here, we exposed the host *Daphnia magna* to two common species of microparasites, targeting distinct tissues within the host and displaying contrasting levels of virulence: a highly virulent, parasitic yeast of the haemolymph (*Metschnikowia bicuspidata*, hereafter referred to as *Metschnikowia*) and a more benign intracellular microsporidium infecting the gut epithelium (*Ordospora colligata*, hereafter referred to as *Ordospora*). Individual *Daphnia* were exposed to either parasite in single infection, simultaneous co-infection or inoculated with a technical control. In addition, these individuals were raised from birth under three concentrations (0, 5 and 50 mg l^−1^) of nano-sized polystyrene plastic beads (100 nm). Based on prior data collected in the *Daphnia*–*Metschnikowia* system, we suspected increasing NP concentrations to improve the infectivity of this fungal parasite, possibly at the detriment of spore production. Considering the intracellular nature of the gut pathogen [51] and the potential for rapid accumulation of NPs in the gastrointestinal tract of *Daphnia* [52], we hypothesized impaired transmission of *Ordospora* under NP exposure. Finally, we assessed whether interactive effects between NPs and parasite co-exposure could further affect the fitness parameters of all three species (i.e. the host and both parasites).

## 2. Material and methods

### (a) Study organisms

The water flea *D. magna* was used as the focal host in this study. One isofemale line (genotype NO-V-7) initially collected in Norway [53] was chosen because of its high compatibility with both parasite strains used in this experiment [48]. *Daphnia* were reared in synthetic culture medium (SSS-medium [54]), at 19°C and 12 : 12 light–dark photoperiod. *Daphnia* were fed three times per week with 1 mg C l^−1^ of *Acutodesmus obliquus,* a green alga maintained in modified Z-medium [55] under a 12 : 12 light cycle at 19°C.

A single strain of the haemolymph yeast *Metschnikowia* (METS_AMME_2008) was isolated from Ammersee, Germany in 2008 and has since been maintained in the laboratory on *D. magna* (genotype E17:07). Similarly, a single strain of the intracellular gut microsporidium *Ordospora* was collected from natural populations of the focal host (*D. magna* NO-V-7) and later maintained in the laboratory on this same genotype. Both parasites can be found in natural *D*. *magna* populations inhabiting shallow ponds and rock pools, where the parasites generally exhibit prevalence of up to 40% (*Ordospora*) and less than 10% (*Metschnikowia*) [42,56]. A generalist parasite of the *Daphnia* genus, *Metschnikowia* spreads via needle-shaped ascospores capable of piercing and crossing the epithelial gut barrier, allowing it to initiate its development and multiplication cycle in the body cavity. Mature asci (i.e. elongated structures containing a single ascospore) are typically observed around 9–10 days following exposure of *D. magna* [48]. An obligate parasite of the species *D. magna*, *Ordospora* establishes and reproduces inside epithelial cells of the digestive tract [51]. Reliable detection of infection symptoms (presence of spore clusters in the gut epithelium or individual spores in crushed host samples) is usually possible starting from day 11 post-exposure [48,57].

### (b) Nanoplastic particles

Spherical polystyrene beads with a nominal diameter of 100 nm (Micromod Partikeltechnologie GmbH, Germany, product code: 29-00-102, product name: micromer-greenF) were stored as a stock solution (10 g l^−1^) at 4°C; for detailed characterization of these particles, see [36]. Two NP concentrations (5 mg l^−1^, 50 mg l^−1^) and a control (0 mg l^−1^) were prepared by dilution in the SSS-medium; these were chosen on the basis of a preliminary survival assay performed with the host (see electronic supplementary material, Appendix S1). NP medium was renewed every fourth day and introduced in the experimental jars 24 h prior to transferring *Daphnia*, to allow for chemical equilibrium.

### (c) Experimental setup and procedures

The experiment consisted of four inoculation treatments: ‘Metschnikowia’ (exposed to *Metschnikowia*), ‘Ordospora’ (exposed to *Ordospora*), ‘Both Parasites’ (simultaneously exposed to *Metschnikowia* and *Ordospora*) and ‘No Parasite’ (exposed to a technical control), as well as three NP treatments: ‘Zero’ (0 mg l^−1^), ‘Low’ (5 mg l^−1^) and ‘High’ (50 mg l^−1^). For each of the resulting 12 combinations, 30 replicates (individual *Daphnia*) were used for a total of 360 experimental units.

On experimental day 1, 360 juveniles born within a 48-h period from synchronized mothers were transferred into individual glass jars filled with 5 ml of medium from their respective NP treatment. Experimental *Daphnia* were fed daily with 1 mg C l^−1^ of *A*. *obliquus* (except the day of parasite inoculation) and maintained at a constant temperature of 19°C under a 12 : 12 light–dark photoperiod. Algal food was harvested three times per week and the appropriate feeding volume was calculated for each new batch, using an established correlation between the optical density (OD 680) and the carbon content of *A. obliquus*. Every fourth day, *Daphnia* were transferred to new experimental jars using autoclaved glass pipettes; this was done to maintain near-constant NP concentrations throughout the experiment (as particle concentration could vary over time due to consumption and internalization by *Daphnia*) and prevent the accumulation of algal food in the medium.

### (d) Parasite inoculation

Parasite inoculation was performed on experimental day 6. Due to unexpectedly high mortality in the ‘High’ NP treatment, as well as some background mortality, experimental *Daphnia* were redistributed prior to the inoculation step to maintain equivalent replication across the intended treatments (see electronic supplementary material, Appendix S2). Replicates from all treatments received two inoculates of equal volume (52.5 µl) containing either 17 300 spores of *Metschnikowia*, 81 375 spores of *Ordospora*, or a technical control containing an equivalent amount of crushed *Daphnia* tissue (genotype NO-V-7). Single infection treatments (‘Metschnikowia’, ‘Ordospora’) received one unit of the respective spore solution and one unit of the technical control; the co-exposure treatment (‘Both Parasites’) received one unit of each parasite's spore solution; the ‘No Parasite’ treatment received two units of the technical control. The final spore concentration introduced for each parasite was 3460 spores ml^−1^ for *Metschnikowia* and 16 275 spores ml^−1^ for *Ordospora*. For further information about the preparation of spore inoculates and the technical control, see electronic supplementary material, Appendix S2.

*Daphnia* were exposed to their respective inoculate for a period of two days, previously shown to induce satisfactory levels of infection with both parasites [48]. Individuals were kept in a relatively low volume (5 ml) during this process to allow for a high density of parasite spores. On experimental day 8, *Daphnia* were transferred to 15 ml of spore-free medium (this final volume was chosen to minimize NP waste production through the remainder of the experiment). From this point onward, *Daphnia* were transferred every fourth day and checked daily for mortality and offspring production (juveniles were counted and removed from the jar). Dead individuals were transferred along with 500 µl of medium from their respective jar into Eppendorf tubes. Samples were fixed with 50 µl of formaldehyde (final concentration: 3.7%) and stored at 4°C until spore yield quantification. The experiment was terminated on day 82; all remaining *Daphnia* were retrieved in the same manner and stored at 4°C.

### (e) Recorded parameters

#### (i) Parasite fitness

Retrieved *Daphnia* were checked individually to detect the presence or absence of parasite spores and quantify the total spore yield per infected host (i.e. *infection intensity*). Samples were blinded within each inoculation treatment, prior to their inspection under the microscope. To quantify the spore yield of *Metschnikowia*, samples were crushed in 0.5 ml and loaded onto a Neubauer Improved counting chamber, after vortexing. Mature, needle-shaped spores were counted under an Olympus SZX16 binocular microscope (115× magnification). To quantify the spore yield of *Ordospora*, samples were crushed in 0.5 ml with 5 µl of a staining agent added to the sample: a solution of Calcofluor-White (1 g l^−1^) mixed with Evans blue (0.5 g l^−1^) was used to stain the chitin-rich walls of microsporidian spores and generate blue fluorescence, to facilitate the counting process [48,58]. Pyriform spores were counted under a Nikon Ti Eclipse inverted microscope, using phase contrast and UV exposure (200× magnification).

All individuals were assigned a binary value for *pre-transmission survival* (0 = *Daphnia* that died prior to day 10 post-inoculation; 1 = *Daphnia* that survived until day 10 post-inoculation, i.e. the earliest timepoint at which spores were detected in crushed individuals). Another binary value was assigned to determine the *infection rate* of either parasite (0 = no spores detected, 1 = spores detected in the sample), among individuals which scored 1 for pre-transmission survival. The *net spore output per exposed host* was used as an assessment of the parasites' overall transmission success. This variable was computed as the effective spore yield recorded per *Daphnia* initially exposed to either parasite: as such, individuals which scored 0 for either *pre-transmission survival* or *infection rate* were included as zero-values, similar to Manzi *et al*. [48]. Additionally, *parasite growth* was determined as the number of spores recorded upon host death, divided by the number of days survived post-inoculation (*infection intensity/host lifespan post-inoculation*; for results, see electronic supplementary material, Appendix S3).

#### (ii) Host fitness

*Host lifespan post-inoculation* was recorded as the number of days survived by individual *Daphnia*, following the inoculation step on day 6. *Host fecundity* was assessed as the total number of parthenogenetic offspring produced per *Daphnia*. The proportion of individuals that produced at least one clutch was also recorded within each treatment (*proportion of mature hosts*; for results, see Appendix S4).

### (f) Data analysis

Statistical analyses were performed in R v.4.1.0 [59]. Graphical outputs were produced using the ‘ggplot2’ [60], ‘ggpubr’ [61] and ‘Hmisc’ [62] packages. Analyses of variance (*F*-test or *χ*^2^ test) were performed with the ‘car’ package [63], using type II sums-of-squares. Individuals that died before the introduction of spore and control inoculates on day 6 were excluded from all analyses, as these could not be attributed to any inoculation treatment. Additionally, due to elevated mortality in the ‘High’ NP treatment, the corresponding data was removed from all statistical analyses. For full disclosure of the collected experimental data, figures incorporating the ‘High’ NP treatment are provided in electronic supplementary material, Appendix S5.

#### (i) Parasite fitness

*Pre-transmission survival* was analysed separately for each inoculation treatment, using a binary logistic regression with ‘NP concentration’ (factor with two levels: ‘Zero’, ‘Low’) as the explanatory variable. *Infection rate* was analysed separately for *Metschnikowia* and *Ordospora*, using a binary logistic regression with ‘NP concentration’ and ‘Inoculation treatment’ (factor with two levels: ‘single exposure’, ‘co-exposure’) as explanatory variables, including their interaction. *Parasite growth* and the *net spore output per exposed host* were also analysed separately for *Metschnikowia* and *Ordospora*, using linear models assuming normal distribution and homoscedasticity of the residuals. The factors ‘NP concentration’ and ‘Inoculation treatment’ were used as explanatory variables, including their interaction. In co-exposure, successfully infected cases were pooled regardless of whether the individual became infected with only the focal parasite or with both parasites. This was done to convey the potential effects of co-exposure *per se* on the recorded variables.

#### (ii) Host fitness

*Host lifespan post-inoculation* and *host fecundity* were analysed using linear models, assuming normal distribution and homoscedasticity of the residuals. The *proportion of mature hosts* was analysed using a logistic regression with the binary scores: 0 = did not reproduce; 1 = produced at least one clutch. Parameters of host fitness were analysed with ‘NP concentration’ and ‘Inoculation treatment’ (here with four levels: ‘Metschnikowia’, ‘Ordospora’, ‘Both Parasites’, ‘No Parasite’) as explanatory variables, including their interaction. As the cut-off necessary for spore detection would lead to an overestimation of host lifespan in the ‘Metschnikowia’, ‘Ordospora’ and ‘Both Parasites’ treatments, individuals whose infection status could not be determined due to dying before day 10 post-inoculation were pooled together with confirmed infected cases. This was done to convey the potential effects of parasite exposure *per se* and improve comparability with the ‘No Parasite’ treatment.

## 3. Results

### (a) Parasite fitness

**Figure 1.**
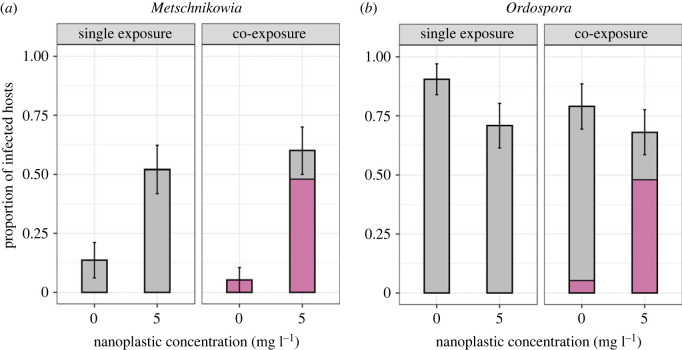
Proportion of confirmed infections with the parasites (*a*) *Metschnikowia* or (*b*) *Ordospora* among hosts that survived at least 10 days post-inoculation. The pink portion of the bars represents the contribution from confirmed co-infections (i.e. spores of both parasites were detected in the sample) to the total number of infections by either focal parasite. Error bars depict the standard error of the mean, computed from a series of binary scores attributed to each individual *Daphnia* (0 = non-infected; 1 = infected). (Online version in color.)

NPs introduced at a low concentration (5 mg l^−1^) had no significant effect on pre-transmission survival of the host (electronic supplementary material, Appendix S3). Infection rates of *Metschnikowia* were four times higher (52% versus 13.6%; ‘single exposure’) and eleven times higher (60% versus 5.3%; ‘co-exposure’) in the ‘Low’ NP treatment, as compared with the ‘Zero’ treatment (figure 1*a* and table 1). There was a tendency towards lower infectivity of *Ordospora* in the ‘Low’ NP treatment, as the infection rate was reduced from 90.5% to 70.8% in single exposure (figure 1*b* and table 1). Infection rates were not affected by the inoculation treatment (i.e. ‘single’ or ‘co-exposure’), and there was no interaction between the two factors (table 1).

**Table 1.** Two-way ANOVA detailing the main effects of the factors ‘NP concentration’ (levels: ‘Zero’, ‘Low’), ‘Inoculation treatment’ (levels: ‘single exposure’, ‘co-exposure’) and their interaction on fitness parameters of the parasites *Metschnikowia* and *Ordospora*. Significant *p*-values (≤ 0.05) are highlighted in italics.

response variable	factor	statistic (d.f.)	*p*-value
*Metschnikowia bicuspidata*			
infection rate	NP concentration	$\chi_{1,87}^{2}=23.164$	*p < 0.001*
	inoculation treatment	$\chi_{1,87}^{2}=0.005$	*p* = 0.942
	NP × inoculation	$\chi_{1,87}^{2}=1.174$	*p* = 0.279
net spore output	NP concentration	*F*_1,105_ = 26.544	*p < 0.001*
	inoculation treatment	*F*_1,105_ = 0.722	*p* = 0.398
	NP × inoculation	*F*_1,105_ = 0.0001	*p* = 0.994
*Ordospora colligata*			
infection rate	NP concentration	$\chi_{1,85}^{2}=2.999$	*p* = 0.083
	inoculation treatment	$\chi_{1,85}^{2}=0.580$	*p* = 0.446
	NP × inoculation	$\chi_{1,85}^{2}=0.518$	*p* = 0.472
net spore output	NP concentration	*F*_1,104_ = 3.499	*p* = 0.064
	inoculation treatment	*F*_1,104_ = 3.689	*p* = 0.058
	NP × inoculation	*F*_1,104_ = 0.590	*p* = 0.444

**Figure 2.**
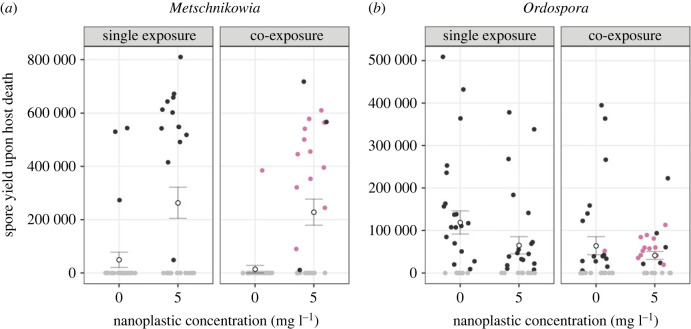
Net spore output of the parasites (*a*) *Metschnikowia* and (*b*) *Ordospora* among all hosts that were exposed to a parasite inoculate on day 6. Black dots represent single infections, pink dots represent confirmed co-infections and grey dots represent individuals that did not yield any spore (included as zero-values). Blank dots depict the mean spore output per exposed host within each treatment, error bars depict the standard error of the mean. (Online version in colour.)

‘Low’ NP concentration strongly increased the net spore output of *Metschnikowia* compared to the ‘Zero’ NP treatment, in which only very few individuals yielded fungal spores (figure 2*a* and table 1). By contrast, the inoculation treatment (‘single’ or ‘co-exposure’) did not influence *Metschnikowia*'s net spore output. Overall, the net spore output of *Ordospora* was marginally decreased under ‘Low’ NP concentration and parasite co-exposure (figure 2*b* and table 1).

### (b) Host fitness

**Figure 3.**
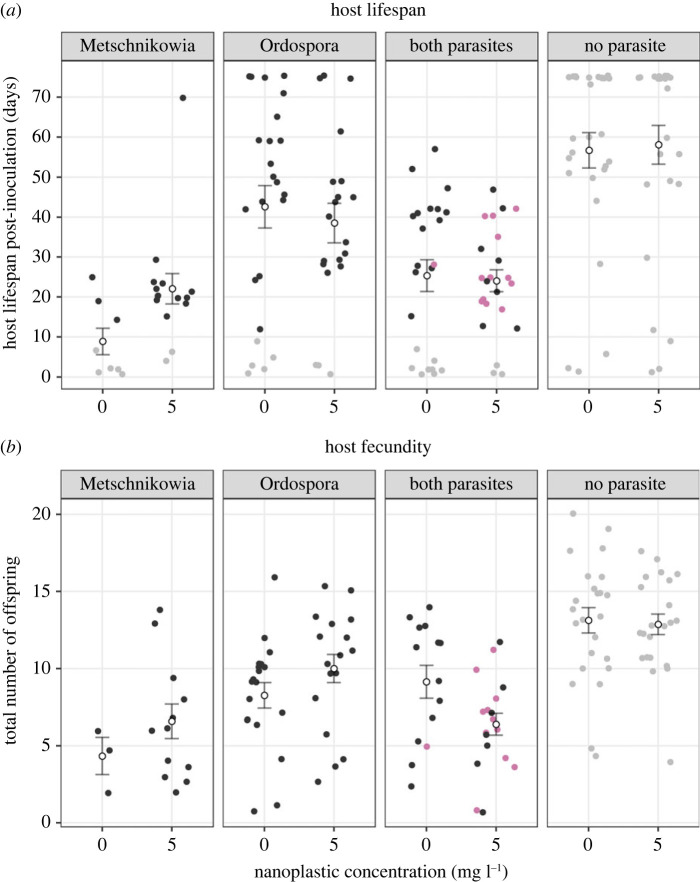
(*a*) Host lifespan (number of days survived post-inoculation) and (*b*) host fecundity (total number of offspring produced by hosts that reproduced at least once) among individuals that were successfully infected, or whose infection status could not be determined due to dying before day 10 post-inoculation (‘Metschnikowia’, ‘Ordospora’, ‘Both Parasites’). These ‘early deaths’ were implemented to improve comparability with the ‘No Parasite’ treatment, as the cut-off necessary for spore detection led to an overestimation of host lifespan in infected cases. Black dots represent single infections, pink dots represent confirmed co-infections, grey dots represent early deaths (‘Metschnikowia’, ‘Ordospora’, ‘Both Parasites’) or control individuals (‘No Parasite’). Blank dots depict the mean lifespan or fecundity within each treatment, error bars depict the standard error of the mean. Individuals which survived past day-10 post-inoculation but did not yield any spore were confirmed ‘non-infected’ and thus excluded. (Online version in colour.)

Introducing NPs at a ‘Low’ concentration in the medium (5 mg l^−1^) did not affect host lifespan. By contrast, there was a significant effect of parasite inoculation on this variable (figure 3*a* and table 2). The average lifespan under *Metschnikowia* exposure was reduced by 23 days compared to the ‘Ordospora’ treatment (*p* < 0.001) and by 39.7 days compared to the ‘No Parasite’ treatment (*p* < 0.001), but did not differ from ‘Both Parasites’ (*p* = 0.56, electronic supplementary material, Appendix S4).

**Table 2.** Two-way ANOVA detailing the main effects of the factors ‘NP concentration’ (levels: ‘Zero’, ‘Low’), ‘Inoculation treatment’ (levels: ‘Metschnikowia’, ‘Ordospora’, ‘Both Parasites’, ‘No Parasite’) and their interaction on fitness parameters of *Daphnia*. Significant *p*-values (≤ 0.05) are highlighted in italics.

response variable	factor	statistic (d.f.)	*p*-value
host lifespan post-inoculation	NP concentration	*F*_1,159_ =0.042	*p* = 0.838
	inoculation treatment	*F*_3,159_ =28.731	*p < 0.001*
	NP × inoculation	*F*_3,159_ =0.836	*p* = 0.476
host fecundity	NP concentration	*F*_1,121_ = 0.034	*p* = 0.853
	inoculation treatment	*F*_3,121_ = 21.151	*p < 0.001*
	NP × inoculation	*F*_3,121_ = 2.547	*p* < 0.059

The total number of offspring produced by *Daphnia* that reproduced at least once was also affected by the inoculation treatment (figure 3*b* and table 2): those exposed to *Metschnikowia* produced 2.7 fewer offspring than the ‘Ordospora’ treatment (*p* = 0.08) and 6.6 fewer than the ‘No Parasite’ treatment (*p* < 0.001), but did not differ from ‘Both Parasites’ (*p* = 0.68; electronic supplementary material, Appendix S4). There was a tendency towards interactive effects of NP exposure and parasite inoculation, as ‘Low’ NP tended to increase fecundity in the single infection treatments (‘Metschnikowia’, ‘Ordospora’) and decrease it with ‘Both Parasites’ (figure 3*b* and table 2). However, pairwise *post hoc* comparisons between ‘Zero’ and ‘Low’ NP were not significant within each inoculation treatment (Tukey's HSD test, electronic supplementary material, Appendix S4).

## 4. Discussion

In nature, *Daphnia* act as mostly non-selective filter-feeders [64,65]. As such, they are prone to ingest not only nutritious food particles (e.g. green algae, heterotrophic bacteria), but also infective propagules of horizontally transmitted pathogens [37] as well as diverse categories of anthropogenically derived contaminants, including NPs [66,67]. Because parasites of *Daphnia* often form plurispecific spore banks in the sediment [42], hosts are also likely to ingest spores of multiple parasites at times. Here, we studied how NPs affect interactions between *D. magna* and two distinct microparasite species, by exposing the host to different concentrations of polystyrene NPs in both single and co-infections. The parasites showcased diverging responses to NP exposure.

### (a) Infection traits of the fungal parasite (*Metschnikowia*) under nanoplastic exposure

Infection rates of the fungal parasite *Metschnikowia* were up to eleven times higher in the presence of NPs, with the strongest effect being detected in co-exposure (‘Both Parasites’ treatment). A putative influence of NPs on infection rates is not surprising, as several mechanisms involved in either parasite encounter or the immune defence of *Daphnia* have been shown to vary under NP exposure. For instance, exposure to MP particles (100–500 nm) resulted in a nearly 30% decrease in feeding rates of *D. magna* [68], while individuals exposed to 100 nm polystyrene beads (similar to the ones used here) also showed a 20% decrease in feeding rates [22]. Additionally, Sadler *et al*. [19] detected an upregulation of haemocyte activity in four out of eight genotypes of *D. magna* exposed to polystyrene MPs (500 nm). As the probability of ingesting parasite spores changes along with foraging rates of *Daphnia* [41,69] and haemocytes constitute the primary defence response against *Metschnikowia* infections [70,71], these typical responses of *Daphnia* do not match with the higher infection rates observed here. Thus, we suspect other processes to be involved in this response, noting that mechanisms of host immunity could have been suppressed instead. Indeed, oxidative stress and genotoxicity (including the suppression of immune-related genes) were both found to be common responses of *Daphnia* exposed to NPs [28,72,73], which may have facilitated fungal infections in this system.

Interestingly, the baseline level of infectivity observed for *Metschnikowia* (i.e. <15% in the ‘Zero’ NP treatment) was much lower than we expected based on a prior infection assay (infection rates ranging from 57% to 74%, after accounting for pre-transmission survival [48]). Instead, the infection rate observed in the ‘Low’ NP treatment was much closer to these expected values. Such a discrepancy in the parasite's infection rate was surprising, considering that we used the same assemblage of host and parasite genotypes, as well as nearly identical inoculation doses (respectively 3460 and 3500 spores ml^−1^). Still, our results indicate that NPs drastically increased the proportion of hosts infected by this fungal parasite. This phenomenon was previously observed in *Daphnia galeata* × *longispina* hybrids exposed to the same strain of *Metschnikowia* at concentrations of 5 and 20 mg l^−1^ polystyrene NPs (S Mavrianos, F Manzi, R Agha, N Azoubib, C Schampera, J Wolinska 2021, unpublished data), suggesting consistency between two host species that typically inhabit different environments. Our results also converge with findings from Li *et al*. [34], who concluded that exposure to NPs in the range of 10–100 µg l^−1^ could enhance infections by parasitic yeast in a *Caenorhabditis* model, by way of suppressed immune response and decreased expression of antimicrobial-related genes.

Considering these results, it begs the question whether this facilitating effect of NPs towards *Metschnikowia* would also apply outside of controlled experimental settings. Seeing as the probability of successful infection of *D. magna* by *Metschnikowia* was multiplied by four in single exposure, this effect seems strong enough to make a difference in nature (although its consistency remains to be tested at lower NP concentrations). As such, future studies implementing field sampling could evaluate whether higher prevalence of *Metschnikowia* can indeed be observed in natural habitats with high concentrations of microplastic and NP debris (though other environmental parameters should be comparable between sites in order to minimize confounding effects). Further, the relatively high virulence and lethality of *Metschnikowia* means that stronger fluctuations in host densities could be expected under NP exposure, possibly implying stronger selection pressure for immune-related loci and mechanisms of parasite avoidance (e.g. habitat choice) in host populations inhabiting highly contaminated sites. Finally, seeing as *Metschnikowia* epidemics have been shown to reduce host density to the point where underlying ecosystem processes can be affected (such as top-down control of phytoplankton biomass [74]), exacerbating effects of NP contamination on this system should be regarded as a potential community-level threat.

### (b) Infection traits of the microsporidium (*Ordospora*) under nanoplastic exposure

Given the possible internalization of NPs inside animal cells [14,15], we suspected the intracellular parasite *Ordospora* to be more susceptible to potential adverse effects of NPs. For instance, NPs ingested by the host could interfere with the internalization process of microsporidian spores inside gut epithelial cells, which involves the formation of a host-derived vacuole [51]. Given that agglutination of NPs in the gut can impair resource acquisition in *Daphnia* [22,75], decreased uptake of microsporidian spores could also derive from reduced foraging activity in the host. Similarly, as microsporidian parasites derive their growth from hijacking intracellular processes of ATP production [76,77], decreased replication of *Ordospora* would likely arise from suboptimal resource allocation in the host.

While we did not find evidence for impaired parasite growth in the presence of NPs (see electronic supplementary material, Appendix S3), our results do indicate lower infection rates in the ‘Low’ NP treatment (decreasing by about 0.20 in single exposure and 0.11 in co-exposure), which led to a marginal decrease in the parasite's net spore output. Such observations tend to converge with the aforementioned predictions on *Ordospora*; however, it should be noted that both effects rely on limited statistical support (table 1). At present, we thus conclude that no facilitating effects of NPs were found on either transmission trait of *Ordospora*, contrary to our observations on *Metschnikowia*. Ideally, future research should investigate how infection mechanisms of *Ordospora* and other gut parasites of *Daphnia* (such as the ichthyosporean *Caullerya mesnili* [45] or the microsporidian *Glugoides intestinalis* [78]) are affected by the accumulation of NP particles in the host's digestive tract.

### (c) Effects of nanoplastics on host fitness parameters

In a previous experimental trial using the same NP particles, *D. galeata × longispina* hybrids displayed a typical reaction of hormesis (characterized by the inducement of an adaptative or beneficial effect on a living organism upon exposure to a low dose of a contaminant [79]) at a concentration of 5 mg l^−1^ (S Mavrianos, F Manzi, R Agha, N Azoubib, C Schampera, J Wolinska 2021, unpublished data). Here, *Daphnia* which were not exposed to parasite inoculates displayed no changes in either their lifespan or offspring production between the ‘Zero’ and ‘Low’ NP treatments. *D. magna* typically shows a higher resistance to contaminants than smaller *Daphnia* species [80], including to polystyrene NPs [81], which may indicate that higher concentrations of polystyrene NPs are also required to trigger the beneficial responses attributed to hormesis, as compared with *D. longispina* complex. In a recent study by Pochelon *et al*. [82], polystyrene NPs with a size of 100 nm (the same that was used in the present experiment) led to the slowest dose–response curve for immobilization of *D. magna*, as compared with smaller size categories of the same particles. Moreover, the maximum concentration tested in that study was 30 mg l^−1^, suggesting that further negative effects could be reached at higher concentrations of polystyrene NPs.

To determine suitable concentrations that could be used in our experiment, we carried out a preliminary trial assessing the survivorship of *D. magna* at concentrations of 5, 20, 50 and 100 mg l^−1^ (see electronic supplementary material, Appendix S1). In this trial, genotype NO-V-7 displayed similar survival at NP concentrations ranging from 0 to 50 mg l^−1^, while elevated mortality only appeared at 100 mg l^−1^. Thus, the extreme levels of mortality that occurred at 50 mg l^−1^ in the main experiment were unexpected. In hindsight, one notable difference between the two protocols consisted of a change in the feeding schedule: *Daphnia* were fed daily with a high concentration of algal food during the main experiment (1 mg C l^−1^), but only every second day during that trial. Thus, we suspect overfeeding may have accentuated either the ingestion or the internalization of NPs in the present experiment, thereby exacerbating their adverse effects.

### (d) Joint effects of nanoplastics and parasite co-exposure on host fitness parameters

One of the motivations behind this study was to test the effects of NPs on two distinct parasites of *Daphnia*. An additional objective was to determine whether NP exposure could sway the outcome of competition in favour of either parasite, and whether interactive effects of NPs with multiple infections would lead to unforeseen levels of damage on host fitness parameters. Overall, we found that the reduction in lifespan and fecundity experienced by co-exposed hosts was generally stronger as compared with single infections by *Ordospora*, but no more than single infections by *Metschnikowia*. This observation proves highly consistent with a previous experimental assay using the same co-infection system [48] and was overall an expected pattern, seeing as the virulence experienced in multiple infection tends to align with the amount of damage induced by the most virulent parasite in *Daphnia* [83–85]. While we did record a reduction in the average fecundity of co-exposed hosts in the ‘Low’ NP treatment, the limited statistical support behind this effect indicates that interactive effects between NP contamination and frequent multiple infections should not be cause for much concern with regards to host populations.

Despite the lack of striking interactions between NP exposure and co-infection on fitness parameters of the host, the association of facilitating effects on *Metschnikowia* with only limited negative effects on *Ordospora* provided an interesting outcome. In accordance with these diverging responses, it appears that successful co-infections had a higher probability of occurring under NP exposure. Notably, when hosts were exposed to both parasites in the absence of contaminant, only 6.7% of all infections by *Ordospora* also had successful transmission of *Metschnikowia*, while 70.6% of hosts infected by *Ordospora* were successfully co-infected under NP exposure. Overall, these observations suggest that NP contamination may have the potential to increase the prevalence of co-infections in natural populations of *D. magna*, despite not aggravating the level of virulence that would be incurred by co-infected hosts.

## 5. Conclusion

We found that the identity of the parasite infecting *Daphnia* strongly determined the influence of NPs on infection outcome. Indeed, exposure to NPs at a concentration that was not sufficient to induce negative effects on the host (5 mg l^−1^) either strongly enhanced (*Metschnikowia*) or slightly hampered (*Ordospora*) infection rates of the parasites, which also reflected on their overall transmission success. Thus, our findings support the idea that distinct species of parasites can show contradictory responses to a given environmental contaminant. While we did not find striking interactive effects between NPs and co-infections, this dichotomy meant that successful co-infections (i.e. exposure leading to the successful transmission of both parasites) also had more chances of occurring under NP exposure. This suggests that NP contamination has the potential to favour parasite coexistence in natural populations of a freshwater keystone species, while possibly exacerbating the effects on population dynamics and further ecosystem functioning derived from increased prevalence of a virulent fungal parasite.

## Data Availability

The data supporting this study can be found at: https://doi.org/10.5281/zenodo.6634688. The data are provided in electronic supplementary material [86].
